# Involved-field radiotherapy for esophageal squamous cell carcinoma: theory and practice

**DOI:** 10.1186/s13014-016-0589-7

**Published:** 2016-02-05

**Authors:** Minghuan Li, Xiaoli Zhang, Fen Zhao, Yijun Luo, Li Kong, Jinming Yu

**Affiliations:** Department of Radiation Oncology, Shandong Cancer Hospital and Institute, Shandong Academy of Medical Sciences, Jiyan Road 440, Jinan, Shandong Province 250117 China; Department of Oncology, Renmin Hospital of Wuhan University, Wuhan, Hubei Province China

**Keywords:** Esophageal carcinoma, Involved-field radiotherapy, Lymph node, Clinical target volume

## Abstract

Esophageal carcinoma (EC) is characterized by a high rate of lymph node metastasis and its spread pattern is not always predictable. Chemoradiotherapy has an important role in the treatment of EC in both the inoperable and the pre-operative settings. However, regarding the target volume for radiation, different clinical practices exist. Theoretically, in addition to the clinical target volume administered to the gross lesion, it might seem logical to deliver a certain dose to the uninvolved regional lymph node area at risk for microscopic disease. However, in practice, it is difficult because of the intolerance of normal tissue to radiotherapy (RT), particularly if all regions containing the cervical, mediastinal, and upper abdominal nodes are covered. To date, the use of elective nodal irradiation (ENI) is still controversial in the field of radiotherapy. Some investigators use involved-field radiotherapy (IFRT) in order to reduce treatment-related toxicities. It is thought that micrometastases can be controlled, to some extent, by chemotherapy and the abscopal effects of radiation. It is the presence of overtly involved lymph nodes rather than the micrometastatic nodes negatively affects survival in patients with EC. In another hand, lymph nodes stationed near primary tumors also receive considerable incidental irradiation doses that may contribute to the elimination of subclinical lesions. These data indicate that an irradiation volume covering only the gross tumor is appropriate. When using ENI or IFRT, very few patients experience solitary regional node failure and out-of-field lymph node failure is not common. Primary tumor recurrence and distant metastases, rather than regional lymph node failure, affect the overall survival in patients with EC. The available evidence indicates that the use of ENI seems to prevent or delay regional nodal relapse rather than improve survival. In a word, these data suggest that IFRT is feasible in EC patients.

## Introduction

Esophageal carcinoma (EC) is a highly lethal disease that has two predominant histological types: adenocarcinoma and squamous cell carcinoma (SCC). These two predominant types may be different diseases, each with a distinct pathogenesis, epidemiology, prognosis and tumor biology, including the pattern of lymph node metastasis [1]. Globally, SCC is the more common histology and the incidence of esophageal cancer is 10 times higher in certain geographic areas in Asia than it is in the U.S., particularly in the “Asian esophageal cancer belt”, which extends from northeast China to the Middle East. Indeed, SCC accounts for 95 % of all ECs in China. Approximately 90 % of all ECs arise in the thoracic esophagus, with the middle thoracic esophagus being the most frequent location [2].

Chemoradiotherapy (CRT) has an important role in the treatment of EC in both the inoperable and the pre-operative settings. Radiotherapy (RT) alone is reserved for palliation or for patients who cannot receive concomitant chemotherapy. Although the International Commission on Radiation Units and Measurements gives suggestions for RT planning, some RT factors are controversial, such as the radiation doses and target volumes. For preoperative therapy, 40–45 Gy is delivered in 1.8–2.0 Gy/day fractions in most studies. For patients undergoing definitive CRT, 50.4 Gy is the accepted standard dose based on randomized data from Europe and North America [3, 4]. Although radiation dose-escalation has failed to improve local control or survival, a dose of 60 Gy is more popular in Asian countries where SCC is the predominant histological type [5, 6]. On the other hand, the target volume of external beam radiation therapy and its coverage are controversial among radiation oncologists. To date, no universally accepted opinion regarding the extent of the RT field has been established, especially for the clinical target volume (CTV). In theory, the CTV includes the gross tumor volume (GTV) in addition to areas at risk of a microscopic metastasis of the disease. For the CTV of a primary tumor, cranial and caudal margins of 3–5 cm and radial margins of 1–1.5 cm are generally used considering submucosal spread [4, 7, 8]. However, for the lymph node CTV, there are various clinical practices. In the era of two-dimensional RT, it has been standard practice to deliver a certain dose to the uninvolved regional lymph node area at risk for microscopic disease. This is known as elective nodal irradiation (ENI). Despite the high risk of nodal spread in EC, the benefit of additional ENI is controversial, especially for overall survival (OS). With the understanding of the biologic characteristics of tumors, technological and chemotherapeutic advances have progressively changed the practice of RT, which has been adopted for treating locally advanced non-small cell lung cancer [9], small cell lung cancer [10], non-Hodgkin lymphoma [11], and Hodgkin lymphoma [12] in the form of involved-field RT (IFRT). New advances in CRT for EC have also come from research into novel ways to minimize toxicity and maximize efficacy. In fact, a few studies [13–17] have used IFRT in definitive CRT or pre-operative CRT for EC patients with either early or locally advanced staged disease. In these studies, all the lymph nodes that were clinically involved were included in the GTV. No prophylactic irradiation was administered to the non-involved regional lymph nodes. However, IFRT is controversial for the increased risk of nodal failure in untreated nodal stations, as clinically uninvolved lymph nodes may harbor microscopic disease.

In this review, the main arguments for omitting ENI in EC patients are discussed, with a particular focus on esophageal SCC. The topics covered include the lymphatic drainage of the esophagus, lymph node micrometastases, the effect of chemotherapy and radiation on micro-metastasis, and the failure patterns and OS after definitive CRT.

### Regional lymph node involvement and the clinical target volume

EC commonly shows lymph node metastases, particularly regional lymph node involvement, which is an early process. For example, the rate of positive loco-regional nodes is close to 0 % for intra-epithelial tumors, 31–56 % for T1b tumors, 58–78 % for T2, 74–81 % for T3, and 83–100 % for T4 tumors in patients with esophageal SCC [18, 19].

For the anatomical lymphatic drainage system of the esophagus, the abundant lymphatic channels in the lamina propria mucosae and submucosa of the esophagus are well known from classic studies. Long longitudinal lymphatic extension in the esophageal submucosa is very evident, and a morphological connection between submucosal lymphatic vessels and recurrent nerve nodes has been observed [20, 21]. The lymphatic routes to the peri-esophageal lymph nodes usually originate from the intermuscular area of the muscularis propria, and lymphatic communication between the submucosa and intermuscular area is rarely apparent histologically [21, 22]. These morphologies suggest an explanation for the anatomically distant lymph node metastasis known as ‘skip metastasis’ observed in EC. Therefore, in patients with tumors confined to the submucosal layer, even with tumors located in the lower esophagus, lymphatic metastasis frequently occurs in the supraclavicular area. The “distant” lymph node involvement from superficial carcinoma is thus not necessarily a sign of advanced disease. In the 7th edition of the TNM staging system, regional lymph nodes were redefined as any paraesophageal lymph node, including the cervical and celiac nodes. For the radiation target volume, theoretically, it might be logical to include not only the mediastinal lymph nodes, but also the cervical and upper abdominal nodes. However, the large radiation volume may increase acute toxicity and late adverse events that involve the heart, lungs, and hematologic system [23]. In the Radiation Therapy Oncology Group trial 85-01, the acute toxicity was substantial: 64 % of patients treated with CRT experienced severe or life-threatening side effects, and only 23 % of patients enrolled were over 70 years of age [24].

To minimize toxicity, tighter fields with less elective CTV coverage (that is, ENI) were used in the later studies, in which the lymph nodes at risk were included in the CTV, depending on the location of the primary tumor. However, there are no universal recommendations for the lymph node CTV for EC. The American National Comprehensive Cancer Network recommends ENI, as follows: for the supraclavicular area, consider treatment of higher echelon cervical nodes, especially if the nodal stage is N1 or greater for cervical lesions; for the proximal third of the esophagus, treat the para-esophageal lymph nodes and supraclavicular areas; for middle lesions, treat the para-esophageal lymph nodes; and for the distal and the gastro-esophageal junction, treat the lesser curvature, celiac axis, and para-esophageal lymph nodes. However, in a recent meta-analysis, Ding et al. [25] surveyed 18,415 patients in 45 observational studies to determine which node level should be included in the target volume for patients undergoing definitive CRT. Their data came from patients undergoing surgical treatment with two-field or three-field dissection. The lymph node metastasis rates of thoracic EC in the cervical, upper-mediastinal, mid-mediastinal, lower mediastinal, and abdominal levels were 30.7, 42.0, 12.9, 2.6, and 9 % for upper thoracic EC; 16.8, 21.1, 28.1, 7.8, and 21.4 % for middle thoracic EC; and 11.0, 10.5, 19.6, 23.0, and 39.9 % for lower thoracic EC, respectively. These results suggest that multiple region involvement and skip node metastasis were frequently observed in EC patients. In theory, a large T-shaped field would be necessary for thoracic EC, which would include bilateral supraclavicular areas, the whole mediastinum, and the left gastric lymph nodes. On the other hand, in addition to the location and depth of tumor invasion, the length of the tumor and histological differentiation were also associated with lymph node metastasis in esophageal SCC [26, 27].

In summary, EC is characterized by a high rate of nodal involvement, and its spread pattern is not always predictable. Although it is thought that there is one predominant area of drainage depending on the location of the primary tumor, it is difficult to accurately define the CTV of RT in a specific patient. It is also difficult to encompass all possible longitudinal local extensions and lymphatic and nodal drainage routes owing to the treatment intolerance of normal tissue.

### Lymph node metastasis and micrometastases

The correct staging of patients with esophageal SCC provides accurate information on the extent of the disease and guides the treatment plan. If IFRT is used, the detection of the involved lymph nodes is important for curative intent. It was reported that, for the detection of regional lymph node metastases, endoscopic ultrasound (EUS) is the most sensitive method, whereas computed tomography (CT) and fluorodeoxyglucose-positron emission tomography (FDG-PET) have higher specificity. The random effects pooled sensitivities of EUS, CT, and FDG-PET for regional lymph node metastases were 0.80 (95 % confidence interval 0.75–0.84), 0.50 (0.41–0.60), and 0.57 (0.43–0.70), respectively, and the specificities were 0.70 (0.65–0.75), 0.83 (0.77–0.89), and 0.85 (0.76–0.95), respectively [28]. In clinical practice, PET-CT is popular for the diagnosis and evaluation of EC. However, there are conflicting results in the imaging literature regarding the accuracy of PET for staging the regional lymph nodes. The major problem is the obscuration of the adjacent lymph nodes by uptake in the primary tumor, leading to low sensitivity. However, PET is useful in detecting regional nodal disease that is not immediately near the primary esophageal tumor. In one study, the sensitivity, specificity, and accuracy of PET-CT were 94, 92, and 92 %, respectively, in a nodal group-by-group basis [29]. Each imaging modality has its advantages and disadvantages; therefore, CT, EUS, and PET should be considered complementary diagnostic methods.

Even so, undetected occult disease recurrence and micrometastases to regional lymph nodes may exist owing to the intrinsic limitation of the accuracy of using all these diagnostic methods. Such micrometastases might be present in a certain number of patients at the time of presentation [30]. In patients with EC, a few studies showed that lymph node micrometastases are associated with a significant negative impact on survival [30–32]. However, some studies have reported that micrometastases might increase the risk of lymph node recurrence, but it does not influence the survival of patients with pN0 esophageal SCC [33, 34].

Although the data of lymphatic mapping of EC indicate that the site of micrometastases is totally unpredictable, it is thought that extensive lymphadenectomy could eliminate the micrometastases to regional lymph nodes. There is evidence from a recent meta-analysis suggesting that a better OS can be achieved with three-field lymphadenectomy *vs.* two-field lymphadenectomy [35]. This may suggest the benefits of ENI. There are no randomized trials investigating the effect of three-field lymphadenectomy in local tumor control and disease-free survival. Nonetheless, some recent reports have suggested that more extensive lymph node clearance during surgery for EC may not improve survival [36, 37]. A few studies have reported that, only a subset of patients will most likely really benefit from extensive lymphadenectomy, which could well be related to the number of lymph nodes involved. Nishimaki et al. [38] reported that no patients with five involved lymph nodes survived for more than 5 years following three-field lymphadenectomy. Rizk et al. also found that patients with more than four involved lymph nodes have survival rates similar to those of patients with M1 disease undergoing surgery [39]. Natsugoe et al. [40] reported that the number of pre-surgical lymph node metastases is related to prognosis in patients with EC, in which the 5-year survival rates of patients with 0, 1–3, 4–7, and ≥8 lymph node metastases determined by US and EUS were 53.3, 33.8 17.0, and 0 %, respectively. These results suggest that the presence of multiple lymph node involvement indicates systemic disease, and that survival remains unchanged despite the extensive removal of lymph nodes. The number of lymph nodes involved is likely more important for survival than the regional field of involvement. Although it has been confirmed that ENI is effective for preventing regional nodal failure [41, 42], for local advanced EC, the micrometastases at distant sites would nullify the benefits of clearance of locoregional disease. There is no doubt that efforts should be made to optimize treatment schedules in order to eliminate distant micrometastases.

These data indicated that the presence of overtly involved lymph nodes rather than the micrometastatic nodes negatively affects survival in patients with EC. The use of intensive radiation and chemotherapy or immune therapy to increase the complete response rate and reduce distant metastases may be essential to improve survival in EC patients.

### Chemoradiotherapy and the incidence of regional nodal failure

Chemotherapy or CRT is able to significantly reduce nodal micrometastases, regardless of whether SCC or adenocarcinoma histology is present [43, 44]. If tumors show a major histomorphologic response following neoadjuvant chemoradiation, the presence of nodal micrometastases is significantly reduced compared to those with a minor response [43]. Tsuchiya et al. [45] reported that preoperative chemotherapy reduced the number of metastatic lymph nodes, and the metastasis rate in the resected lymph nodes was significantly higher in patients who underwent surgery alone than in those who received preoperative chemotherapy. Furthermore, the mean number of metastatic lymph nodes was significantly lower in the chemotherapy responders than in non-responders. In responders, tumors exhibited such a high degree of CRT sensitivity that not only the primary tumor, but also distant micrometastases, were eliminated by the neoadjuvant therapy. It is possible that, in the patients with a partial response (PR) and with variable tumor sensitivity, the persistence of distant micrometastases caused distant tumor recurrence. In an aggressive cancer, any remaining distant micro-metastasis after neoadjuvant therapy ensured that patients with a PR experienced recurrence outside of the treated field with the same virulence as those non-responding patients not affected by neoadjuvant therapy.

In contrast, the clinical relevance of micrometastases may depend on the balance between tumor aggressiveness, the host immune status, and the response to treatment. Theoretically, a host’s immune system may be able to remove a single tumor cell and diminish residual cells with metastatic potential. Mounting evidence indicates that RT also recruits biological effectors outside the treatment field and has systemic effects [46, 47]. When tumor cells die due to RT, they also emit a specific combination of signals that elicits tumor-specific cytotoxic T lymphocyte responses. The immune effectors that are generated in this setting can act systemically, hence eradicating distant, non-irradiated lesions (long-range, out-of-field or abscopal effects). In immunocompetent hosts, the efficacy of RT appears to rely for the most part on abscopal effects. If the tumor is sensitive to radiation, to some extent, IFRT could also significantly reduce nodal and distant micrometastases.

Thirdly, incidental irradiation may play a role in the control of micrometastases in thoracic lymph nodes. Although the use of intensity-modulated RT and IFRT can reduce the incidental irradiation of the regional nodal area, irradiation around the primary lesions may have still been effective in eradicating subclinical lymph node metastasis. For the primary tumor, radiation fields extended 3–5 cm beyond the proximal and distal extent of the lesion, and the lateral borders extended 1–2 cm beyond the apparent mass in most studies. As such, the periesophageal lymph nodes should be included in the radiation field. Ji et al. [48] showed that that the incidental irradiation dose to high-risk nodal regions is considerable for T1-3N0M0 esophageal SCC using three-dimensional CRT. Based on a meta-analysis by Ding et al. [25], lymph node metastasis most frequently occurred in paratracheal, paraesophageal, perigastric, and subcarinal stations. In clinical practice, however, most patients presented with long lesions and/or positive lymph node metastasis, in which more high-risk regions would receive considerable doses if metastatic nodes were included in the GTV. Therefore, IFRT may still deliver a considerable incidental dose to high-risk elective regions, which will have a significant impact on the control of micrometastases.

In conclusion, it is thought that, to some extent, micrometastases can be controlled by chemotherapy and the abscopal effects of radiation in responders. For non-responders, the persistent gross disease and the distant micrometastases cause the immediate recurrence, which may mask regional nodal failures. On the other hand, lymph node stations near primary tumors also receive considerable incidental irradiation doses that may contribute to the elimination of subclinical lesions. These data indicate that an irradiation volume covering only the gross tumor is appropriate.

### Patterns of failure

The response rate decreases with the progression of disease. Many trials have reported a good response rate from concurrent CRT in esophageal SCC [3, 4, 16, 49–56]. Based on these data, the response rate by stage was more than 90 % for T1, 60–90 % for T2–3, and 57–88 % for T4. The complete response (CR) rate by stage was 89.7–97 % for T1, 50–60 % for T2–3, and 17–39 % for T4, respectively. The response status is well correlated with the failure patterns. Rohatgi et al. demonstrated that the proportion of residual carcinoma after preoperative CRT is significantly correlated with the patterns of locoregional and distant failure [57]. Similar results were also found in patients receiving definitive CRT for EC [58]. The time to locoregional recurrence was significantly longer for patients who achieved a pathologic complete response compared with non-responders. The rate of distant metastases was significantly lower in responders compared to non-responders.

For T1 stage disease, most patients achieved a CR after definitive (C)RT. Nonetheless, half of the failures occurred in the local primary tumor, and the secondary failure pattern was the distant lymph nodes or organs [14, 16, 49]. The incidence of regional lymph node failure is low (1–6 %), with or without ENI. Ishikawa et al. assessed the patterns of failure after IFRT in 68 patients with stage I EC. In their study, out of 50 patients with submucosal cancer (T1b), only 1 patient (2 %) developed regional lymph node failure outside the radiation field [59]. Okawa et al. conducted a multi-institutional study of 105 patients with superficial EC treated with ENI. Lymph node failure outside the radiation field occurred in 6 patients (6 %), whereas lymph node failure inside the ENI area occurred in only 1 patient (1 %) [60]. Kawaguchi [16] also observed regional lymph node failure alone in 4 % (3/68) of patients with clinical stage I thoracic esophageal SCC after IFRT. In contrast, for early stage esophageal SCC, a second primary cancer was often observed. In the JCOG9708 study, a second primary cancer was observed in 18 of 72 patients [49]. Yamamoto et al. also found that most local recurrences in the CRT group were intramucosal metachronous carcinomas for the stage I population, and they were cured after salvage treatment with no effect on OS [61]. As such, the incidence of regional lymph node failure is low, which suggests that conservative IFRT should be feasible for T1 stage EC.

In locally advanced patients with SCC histology, CRT generally results in CR rates of ∼ 20–50 %. Unfortunately, a considerable proportion of EC patients were resistant to CRT, and as many as 11–26 % of those patients did not exhibit any morphological response, which results in persistent disease or immediate local failure. Welsh et al. reported the results of failure patterns in patients with EC treated with definitive CRT using ENI [62]. At a median follow-up time of 52.6 months, 50 % had experienced local failure, 48 % had distant failure, and 31 % had no evidence of failure. Of all local failures, 90 % were within the GTV, 23 % were within the CTV, and 12 % were within in the planning target volume. However, the histological type was adenocarcinoma (76 %) rather than SCC (24 %) in that study. In a report by Versteijne et al. [63], in which half (52 %) of the patients had SCC, the conformal CTV consisted of the GTV plus at least the periesophageal lymph node area extended in the cranio-caudal direction by a 3.5-cm margin. With a mean follow-up time of 22.8 months, 41 % of patients had evidence of a locoregional recurrence. The median time to locoregional recurrence was 9.6 months. Among the patients with a locoregional recurrence, most showed failure at the site of the GTV (86 %). A failure at the site of the primary tumor alone occurred in 57 %, in lymph nodes alone (in- and outfield) in 14 %, and at both sites in 29 %. Of the total group of 184 patients, 76 patients (41 %) experienced distant metastases, of which 37 (20 %) occurred in combination with a locoregional recurrence. In this study with reduced ENI, the out-field locoregional recurrences occurred in 17 of 184 patients (9.2 %). In 8 of these patients (4.3 %), it was a solitary locoregional recurrence without an infield recurrence, which is comparable with the results observed with ENI. In another IFRT study based on FDG-PET staging for inoperable esophageal SCC with lymph node metastases, Yamashita et al. [17] found only 2 of 63 patients with failure in lymph node regions not included in the target volume.

For patients received preoperative IFRT, Oppedijk et al. reported a 1 % (2/213) failure rate for isolated out field locoregional recurrence in the CROSS trials. Though locoregional recurrence occurred in 6 % outside the radiation target volume, most of them (11/13) combined with distant failure in this trial [64]. Even for cases with a CR after CRT, the primary lesion and the distant organ rather than the regional lymph node were the prominent sites of recurrence. In a study by Di Fiore et al. [65], the target volume of RT was the macroscopic tumor and enlarged lymph nodes. For the 86 clinical CR patients, 34 (39.5 %) experienced a local disease recurrence, 37 patients (43 %) experienced metastatic disease, and 19 of them experienced both of these. Using ENI combined with chemotherapy, Yamashita et al. [41] found that, after achieving a CR, 40 patients experienced failures (local failure in 20 and distant failure in 20), and no patient experienced elective nodal failure without having any other site of recurrence. Onozawa et al. [42] reported failure patterns using ENI for the 20 patients with recurrence after a CR; the first sites of failure were local (10 patients), distant (9 patients), and elective nodal failure (1 patient). Of note, the vast majority (80 %) of the patients with in-field lymph node pathology had a synchronous failure at the primary tumor site. It may be hypothesized that part of this lymph node metastasis was secondary to failure at the site of their primary tumor.

In conclusion, for early and advanced EC, with either ENI or IFRT, the majority of the failures occurred in the GTV (especially in the primary tumor) and at distant sites after CRT, even for cases with a CR. The lymph node failure in or out of field was not common, and only few patients experienced solitary regional node failure.

### Overall survival

According to trials of local or advanced EC treated with CRT using ENI, 1-year OS rate was 41–88 %, 2-year OS was 28–63 %, 3-year OS was 19–48 %, and 5-year OS was up to 26 %; the median survival time was 9.0–33 months, depending on the disease stage [3–5, 50–53]. In an IFRT study using FDG-PET, Yamashita et al. [17] showed that the 3-year progression-free survival (PFS) and OS rates were 47.7 and 51.1 %, respectively, with a median PFS and OS of 34.6 and 38.4 months, respectively. In another IFRT study, Zhao et al. [13] found that the median survival and PFS were 30 months and 17 months, respectively, with 1-, 3-, and 5-year OS and PFS rates of 77, 56, 41, and 77, 55, 36 %, respectively. For patients aged 75 years and older, Uno et al. [66] demonstrated that they could also benefit from IFRT combined chemotherapy, and a CR was obtained in 6 of 22 patients (27.3 %), with a median survival time of 9 months and a 1-year OS rate of 39 %. These data were comparable with those of the ENI studies. Ma et al. [67] directly compared ENI with IFRT in patients with cervical and upper-thoracic EC and found a median survival time of 33.7 months for the IFRT group versus 32.7 months for the ENI group in a prospectively randomized trial. However, no significant difference was found in the 3-year OS and local-regional control rates between the IFRT and ENI groups (32.0 and 80.1 % *vs.* 41.3 and 85.7 %, respectively). In a JASTRO study [68], they found that there was no difference between the survival rates of patients who had received RT to the primary lesion alone and those of patients who had received RT to the primary lesion and regional lymph nodes in either patients with mucosal or submucosal cancer.

Regarding the impact of regional lymph node failure on OS, there have been few studies comparing IFRT and ENI. Liu et al. [69] retrospectively evaluated the value of ENI for cervical and upper thoracic EC, and they found that out-of-field regional cervical node metastasis occurred in 8 % of patients in the IFRT group. However, it occurred in 10 % of patients in the ENI group. ENI for cervical and upper thoracic esophageal SCC patients did not lead to longer OS or better long-term control of cervical lymph nodes. Although ENI might delay cervical node progression in the elective field, it does not decrease the incidence of these failures. Hsu et al. [70] retrospectively analyzed the of outcome differences in preoperative concurrent CRT with or without ENI for esophageal SCC. They found that more patients in the non-ENI group had M1a (cervical or abdominal regions) failure than in the ENI group, with 3-year rates of 11 and 3 %, respectively (*p* = 0.05). However, the M1a failure was not associated with poor outcomes in patients undergoing preoperative CRT for esophageal SCC. Matched cases analysis did not show a statistical difference in the outcomes between the groups. Zhang et al. [14] observed the contribution of different failure patterns to survival. In their study, the median OS time for patients with in-field failure was 14.2 months *vs.* 17.4 months (*p* = 0.010) for those with non in-field failure. Patients without distant failure achieved a better OS than those with distant failure (16.2 m *vs.* 13.2 m. *p* < 0.001). However, no significant difference was found in the median OS time for patients with or without an out-of-field regional lymph node failure pattern.

While OS should remain a primary endpoint, treatment-related toxicity should also be considered. ENI studies reported that 25–60 % and 23–29 % of patients experienced grade 3 or greater acute and late toxicities, respectively [3, 4, 24, 71]. In Kaneko’s study [50], which used extended field of irradiation and concurrent chemotherapy for patients with malignant strictures of esophageal carcinoma. Grade 3 and above leukocytopenia, anemia, thrombocytopenia, and esophagitis occurred in 30, 33, 14, and 25 % of patients, respectively. Zhao et al.[13] reported rates of grade 3 acute and late toxicities of 9 and 6 % after IFRT, respectively, and no patients had acute or late grade 4 or 5 toxicities. The patient compliance and tolerance is well when IFRT was used in the trial FFCD 9901 [72]. In group CRT, 91.8 % patients received a total radiation dose, with 92.9 % patients completing the first cycle of chemotherapy and 85.7 % completing the second cycle. During the first and second cycles of chemotherapy, 14.3 and 13.3 % patients experienced grade 3 or 4 toxicities, respectively. There were no treatment-related deaths before surgery. Ma et al. [67] also showed that there were significant less severe in the IFRT group than ENI groups regarding hematologic toxicity, infection and vomiting. Thus, IFRT may reduce incidences of treatment toxicities and enable more patients to tolerate chemoradiotherapy.

These studies indicate that primary tumor recurrence and distant metastasis, rather than regional lymph nodal failure, affect the OS in patients with local advanced EC. No significant difference was found in the OS of patients treated with ENI or IFRT and IFRT may reduce incidences of treatment toxicities, which suggests that IFRT is the rational choice.

## Conclusion

In conclusion, the GTV was the most common site of initial failure after CRT in EC patients, and advanced-stage patients experienced high rates of systemic failure. It remains unclear how much of the potential improved OS with the addition of ENI is caused from the improved regional tumor control. It is reasonable that only patients with a histologically proven CR in terms of GTV can acquire PFS benefits from eliminating the regional nodal micrometastases. However, the responders also have fewer incidences of any patterns of failure compared to non-responders. Consequently, the role of ENI in the prevention of regional failure was also challenged in patients with good responses. In addition, for non-responders or non-complete responders, if the primary lesions could not be well controlled, ENI seems unnecessary. Although ENI should be able to decrease the regional nodal failure, non-responders have shorter survival, and many of the patients died before their regional nodal failure became clinically apparent or threatened their life.

In a word, these data suggested that IFRT is feasible in EC patients. The use of intensive radiation and chemotherapy to increase the complete response rate and reduce distant metastases may be essential to improve survival. Future research also should improve our ability to increase the diagnostic accuracy of metastatic lymph nodes, predict the risk of lymph node involvement, and identify responders prior to treatment. Validation of this opinion by future prospective and randomized studies is, however, required.

## Abbreviations

3D-CRT: three-dimensional conformal radiotherapy
CR: complete response
CRT: chemoradiotherapy
CT: computed tomography
CTV: clinical target volume
ENI: elective nodal irradiation
EUS: endoscopic ultrasound
FDG-PET: fluorodeoxyglucose-positron emission tomography
GTV: gross tumor volume
IFRT: involved-field radiotherapy
OS: overall survival
PFS: progess free survival
PR: partial response
PTV: planning target volume
RT: radiotherapy
SCC: squamous cell carcinoma
